# Impact of mesoscale eddies on chlorophyll variability off the coast of Chile

**DOI:** 10.1371/journal.pone.0203598

**Published:** 2018-09-13

**Authors:** Yuntao Wang, Hao-Ran Zhang, Fei Chai, Yeping Yuan

**Affiliations:** 1 State Key Lab of SOED, Second Institute of Oceanography, Hangzhou, China; 2 College of Oceanography, Hohai University, Nanjing, China; 3 School of Marine Sciences, University of Maine, Orono, ME, United States of America; 4 Ocean College, Zhejiang University, Zhoushan, China; Universidade de Aveiro, PORTUGAL

## Abstract

The mesoscale eddies off the coast of Chile significantly impact the distribution of local chlorophyll and the development of marine ecosystem. Multiple processes, including eddy trapping, pumping, advection, Ekman-pumping, and submesoscale dynamics, exert their impacts simultaneously on transport of water masses at different distances with respect to the eddy center. The cyclonic (anticyclonic) eddies are generally characterized by upwelling (downwelling) within the eddy, which elevates (depresses) chlorophyll inside the eddy. Outside the eddy periphery, multiple processes are involved simultaneously, but their corresponding influences on chlorophyll are not well identified. In this study, the amplitudes of cyclonic and anticyclonic eddies are distinguished as positive and negative values, respectively. A linear regression method is applied to seek the connection between eddy’s amplitude and chlorophyll distribution at different locations w.r.t. the eddy center. The regression slope between eddy amplitude and chlorophyll anomaly is found to be negative in the eddy interior and along the periphery, which gradually changes to positive away from the periphery. The location where the response of chlorophyll to an eddy switches its sign is defined as the transition zone. The location of the transition zone varies with offshore distance and is impacted by topography, such as the presence of islands, which can change the dynamics of eddies. Thus, the distance from eddy center and offshore distance from coast should be taken into consideration when investigating their influences on nutrient transport and chlorophyll distribution.

## 1. Introduction

Mesoscale eddies are ubiquitous features in the global ocean and can occupy 25–30% of the total ocean surface [1]. These eddies generally propagate westward at speeds similar to the phase speeds of long baroclinic Rossby waves [2]. The eddies are characterized by nonlinear features that the rotating speed of an eddy is larger than its phase speed, so they can transport energy and nutrients associated with them [3]. Chelton [2] detected global eddies using altimetry data and found the Eastern Boundary Current Systems were particularly favorable for the generation of eddies. This was because of geometry around islands [4], coastline curvature [5], meandering of boundary currents [1], coastal upwelling-induced offshore transport [6], among others.

Eddies are highly dynamic, involving different processes that can happen simultaneously at different distances with respect to the eddy center. Based on the physical characteristics of the eddy, these processes influence the water exchange in horizontal direction [7] and vertical direction [8], regardless the eddy is in the southern hemisphere [9, 10] or the northern hemisphere [11]. And this vertical water exchange can reach deeper than the mixed layer [8, 12]. Therefore, these processes can influence the growth of phytoplankton by transporting nutrients between eutrophic layer and deeper layer [13, 14]. For the region inside an eddy, eddy pumping [15, 16], eddy trapping [7] and eddy-Ekman pumping [17] are dominating processes that influence the distribution of surface chlorophyll. During eddy intensification, the eddy pumping induced upwelling results in positive chlorophyll anomalies inside a cyclonic eddy [18, 19] and downwelling results in negative chlorophyll anomalies in an anticyclonic eddy [20, 21]. The water trapped inside an eddy during its formation can be preserved within the eddy as it propagates away from its origin [22, 23]. The coastal water is higher in phytoplankton comparing with the offshore water [24]. The eddies induced by meanders can trap water with elevated chlorophyll as they pinch off the main current [21]. When wind blows over an eddy, wind stress is intensified or weakened at the side where eddy rotates against or along the wind direction, respectively [14, 25]. The eddy-induced non-uniform wind stress will lead to Ekman transport with different strength [26]. The non-uniform Ekman transport, named eddy-Ekman pumping [27], will induce vertical transport and chlorophyll anomalies opposite to the eddy pumping. However, the magnitude of transport by eddy-Ekman pumping is comparably weak [17, 25].

Along the eddy periphery, chlorophyll distribution is largely influenced by eddy advection, and higher chlorophyll can be found near the periphery. In regions with strong horizontal chlorophyll gradient, eddies will stir the chlorophyll field and advect low chlorophyll water into high chlorophyll area or reversely [3]. The chlorophyll anomalies induced by eddy advection can generate asymmetric dipoles [3]. Guo [28] found advection became the largest at the eddy periphery. Consistently, elevated chlorophyll was observed at the eddy periphery [29]. Mizobata [30] found high chlorophyll concentration near the periphery of an anticyclonic eddy because nutrient-rich deep water inside the eddy was transported along the upward displaced isopycnals to the surface near the eddy periphery.

Outside of eddies, the eddies induce submesoscale structures that can drive vertical mixing and transport [31]. The upwelling can induce significant amount of chlorophyll beyond the anticyclonic periphery [32]. However, detailed mechanism for submesoscale dynamics are not well understood yet. A dynamic structure of pressure anomaly for eddies can be described using a function of radial distance. The pressure anomaly outside of the eddy core has the opposite sign comparing with that inside [33].

Besides the distinct dynamic processes at different distances with respect to the eddy center, the eddies vary largely by their polarity, location and feature [2]. Indeed, the impacts of cyclonic and anticyclonic eddies are usually discussed separately [20, 34]. For example, Dufois [13] suggested that convective mixing was enhanced in anticyclonic eddies of the Indian Ocean, which could inject nutrients into the mixed layer and lead to elevated surface chlorophyll. But corresponding pattern was not observed for cyclonic eddies. Eddy amplitude is one of the important features that could be used to quantify the influence of eddy. Eddy pumping-induced vertical flux of nutrients into the euphotic zone is linearly related to eddy amplitude [18], and strong correlation has been found between chlorophyll anomaly and eddy amplitude [35].

Despite intense studies that have been carried out to investigate the dynamics for mesoscale eddies, the influences of mesoscale eddies on chlorophyll distribution at different spatial scales remain unclear. Previous studies mainly described eddy-induced chlorophyll anomaly as one single number by averaging chlorophyll field over the entire eddy or described the distinct spatial features between cyclonic and anticyclonic eddies separately. More recently, the size of phytoplankton cells was found to change with seaward distances respecting to eddy center [36]. In this study, we focused on the region off the coast of Chile. The region was identified as the highest fisheries production in the world [1] due to coastal upwelling and eddy activities [6], among others. Model results showed upwelling in cyclonic eddies, originated from the Peru Undercurrent [37], could supply nutrients for the development of marine ecosystem. As eddies can impact sea level, thus negative cross correlation between sea level anomalies and chlorophyll anomalies was found off the coast of Chile [21]. By analyzing the chlorophyll distribution associated with eddies, we discovered important spatial variation of chlorophyll from eddy core to its surrounding. Besides the polarity of eddies, the location of eddies should be taken into consideration when investigating their influences on nutrient transport and chlorophyll distribution. The data and method are described in section 2. Our major findings are summarized in section 3. Possible mechanisms for what are discussed in section 4, following by summary and future study plan in section 5.

## 2. Data and method

The mesoscale eddies in this study were identified by Chelton [2], using the AVISO altimeter dataset with 25-km spatial resolution. The data in the regions less than 50 km from the coast were discarded to avoid land contamination. Briefly, an eddy was idealized as a circle with centroid identified at local minimum (maximum) of sea surface height (SSH) for cyclonic (anticyclonic) eddy. The circle covered the same area with the largest enclosed contour with SSH value below (above) a given threshold. The radius of the eddy, *R*, was subsequently calculated based on the circle size:

R=Sπ

where *S* represented the area of the eddy. Eddy periphery was defined as the boundary of eddy centered at the centroid with calculated radius. To distinguish different location with respect to the eddy core, the ratio between the radius of concentric circle and the radius of the eddy was defined as RDR (Ratio of Distance from eddy center to corresponding eddy Radius). The amplitude of cyclonic (anticyclonic) eddy was defined as the difference between the minimum (maximum) SSH within the eddy and the average SSH along the eddy periphery. Thus, the amplitude for cyclonic (anticyclonic) eddy was negative (positive). Eddies were detected globally for each day as snapshots, and their tracks were identified for individual eddies. Detailed procedures for eddy detection can be found in [2].

The chlorophyll data were obtained from the Moderate Resolution Imaging Spectroradiometer (MODIS) onboard NASA’s satellite. The temporal coverage for chlorophyll used in this study is between 2003 and 2014 at daily interval. The spatial resolution of chlorophyll observations is 4.5 km, and the first pixel next to land was discarded to reduce the impact of land [6]. The data were log-transformed because of their log-normal distribution [38]. The chlorophyll data were then averaged using three-day running mean to reduce the impact of cloud coverage. To investigate the responses of chlorophyll to eddies, chlorophyll observations were compared with eddies identified on the same day. The anomaly field of chlorophyll observations was calculated as the deviation from an area mean field of 600 km×600 km [19, 39], centered at each grid. This helped to remove large-scale background signals without altering mesoscale features. Different smoothing and filtering methods had been tested, and the results were statistically identical; thus, the corresponding results will not be reported here.

Instead of calculating the overall average of chlorophyll anomaly for each eddy, we decomposed eddies into different concentric rings from eddy centroid up to RDR = 1.7 and the width of each ring spans 0.3 RDR. The RDR of concentric ring was defined as the RDR at the center of the ring. For example, the concentric ring with 1.35 RDR represented the ring with an inner circle that had a RDR of 1.2 and the outer circle that had a RDR of 1.5. When the RDR of concentric ring was less (more) than 0.85 (1.15), it was referred to as inside (outside) of the eddy. The periphery was inside the concentric ring if the RDR was between 0.85 and 1.15. The mean of chlorophyll anomaly was subsequently calculated for each concentric ring. The influence of eddy on chlorophyll variability was then quantified by the correlation coefficient between binned eddy amplitude and corresponding mean eddy chlorophyll anomaly for concentric rings [27]. Eddies affected by more than 50% overall cloud coverage of chlorophyll observation were excluded in this study.

## 3. Results

**Fig 1:**
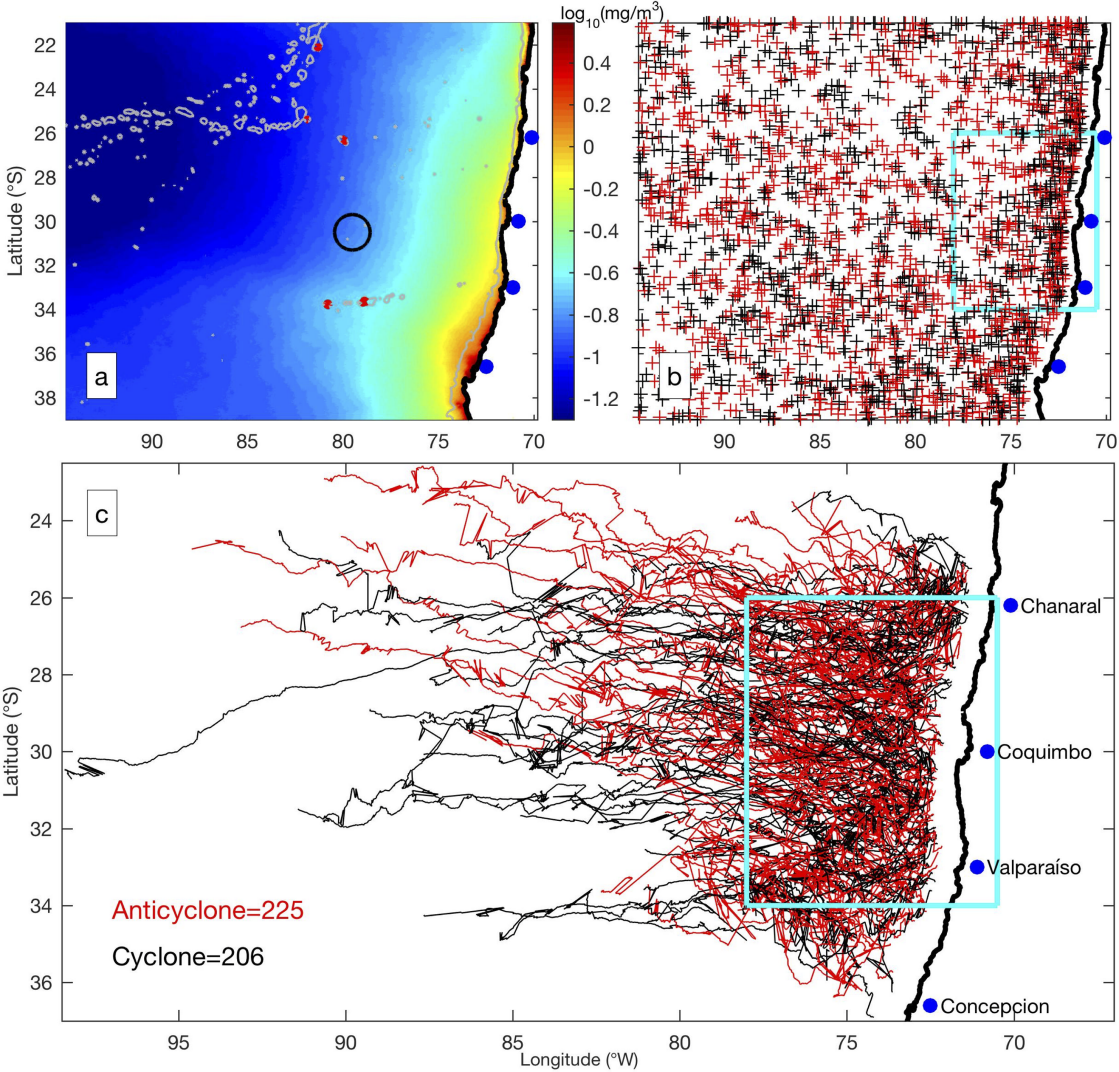
Study region and general features. (a) Average of chlorophyll (in logarithm scale) off Chile with 2000-m isobath indicated by grey contour. Various islands are marked by red dots. The black circle indicates the region for conducting illustration in Fig 3. (b) Initial locations for cyclonic (black) and anticyclonic (red) eddies when they are first detected. (c) The trajectories of eddies originated within the cyan box. The trajectories of cyclonic and anticyclonic eddies are plotted in black and red, respectively. The total numbers of eddies are given at the lower left corner.

There were 332,835 snapshots of eddies from 2003 to 2014 in our study area (70°W-94°W, 21°S-39°S), corresponding to 2,536 individual eddies. Statistical features of these eddies are given in Table 1. The trajectories of the eddies showed that both cyclonic and anticyclonic eddies mainly propagated westward away from the coast (Fig 1C), consistent with the general features of global eddies [2]. Regions with offshore distances between 80 and 300 km were favorable for the formation of eddies, especially at the lee side of a cape (Fig 1B). The distribution of chlorophyll was an important factor for identifying primary production off the coast of Chile [40]. The mean chlorophyll distribution was higher near the coast and decreased offshore (Fig 1A). Chlorophyll was also higher around islands, e.g., the Juan Fernández Islands (near 80°W, 33°S). The meridional gradient of chlorophyll was much less pronounced.

**Table 1:** Eddy features off chile (70°W-94°W, 21°S-39°S) during 2003–2014.

Eddy features	All Eddies	Cyclonic Eddies	Anticyclonic Eddies
Number of eddies	2,536	1,203	1,333
Number of snapshots	332,861	159,271	173,590
Mean amplitude (cm)	3.97 ± 2.12	4.06 ± 2.13	3.89 ± 2.11
Mean radius (km)	81.7 ± 26.1	82.3 ± 25.9	81.2 ± 26.3
Mean lifetime (days)	131.2 ± 154.8	132.4 ± 148.3	130.3 ± 160.4
Mean rotating speed (cm/s)	10.6 ± 3.6	10.9 ± 3.8	10.4 ± 3.3

The standard deviations for amplitude, radius, lifetime and rotating speed are included.

**Fig 2:**
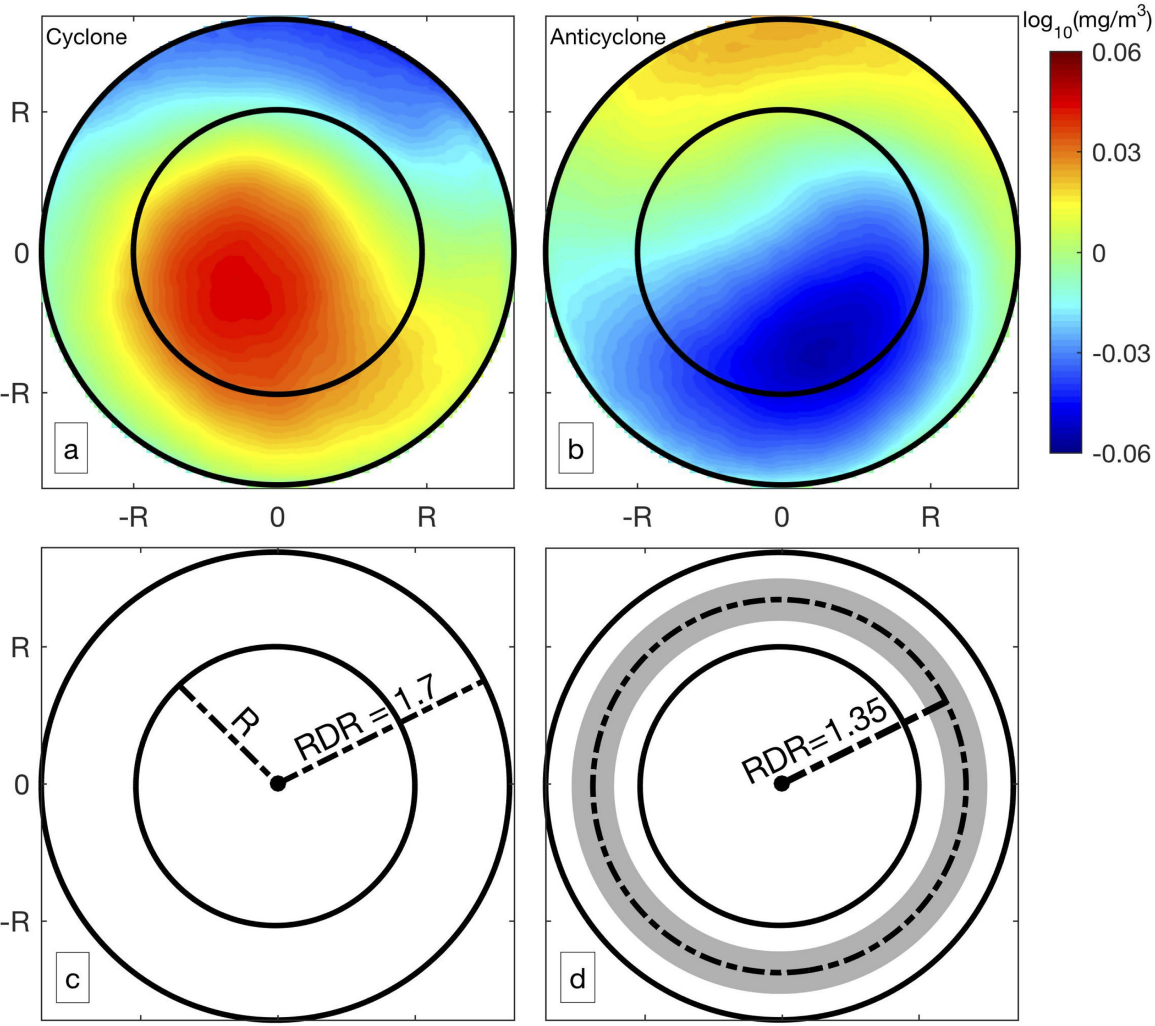
Average chlorophyll anomaly for composite eddies and description of RDR. Composite cyclonic eddy (a, total number of snapshots = 68,399) and anticyclonic eddy (b, total number of snapshots = 68,159). The description for RDR equals to 1.7 is shown in (c) where the circle with radius equals to R is the boundary of the eddy. The chlorophyll anomaly field was calculated for concentric ring as illustrated by shading in (d). The ring has RDR equals to 1.35, and the width of each band is 0.3 times RDR.

The impact of eddies on the distribution of chlorophyll was investigated by using chlorophyll anomaly near each snapshot of eddy. Concerning the large difference among eddies, cyclonic and anticyclonic eddies were collocated with corresponding distributions of chlorophyll anomaly to generate composite eddies, respectively. All valid observations of chlorophyll (with cloud coverage less than 50%) with RDR less than 1.7 for cyclonic and anticyclonic eddies were collocated and interpolated into the same scale for generating composite eddies (Fig 2A and 2B). Chlorophyll anomaly with positive value occupied more than 70% of the cyclonic eddy area, especially in the southwestern section. Negative Chlorophyll anomaly was much weaker and occupied small area in the northeastern section. For anticyclonic eddies, positive (negative) Chlorophyll values were found in the northern (southeastern) section. Negative Chlorophyll anomaly occupied majority of the composite eddy, especially the southern half of the study area. The chlorophyll distribution varied apparently with the distance from the eddy center, and the anomaly field was evaluated by using concentric ring from the eddy core (Fig 2C and 2D).

**Fig 3:**
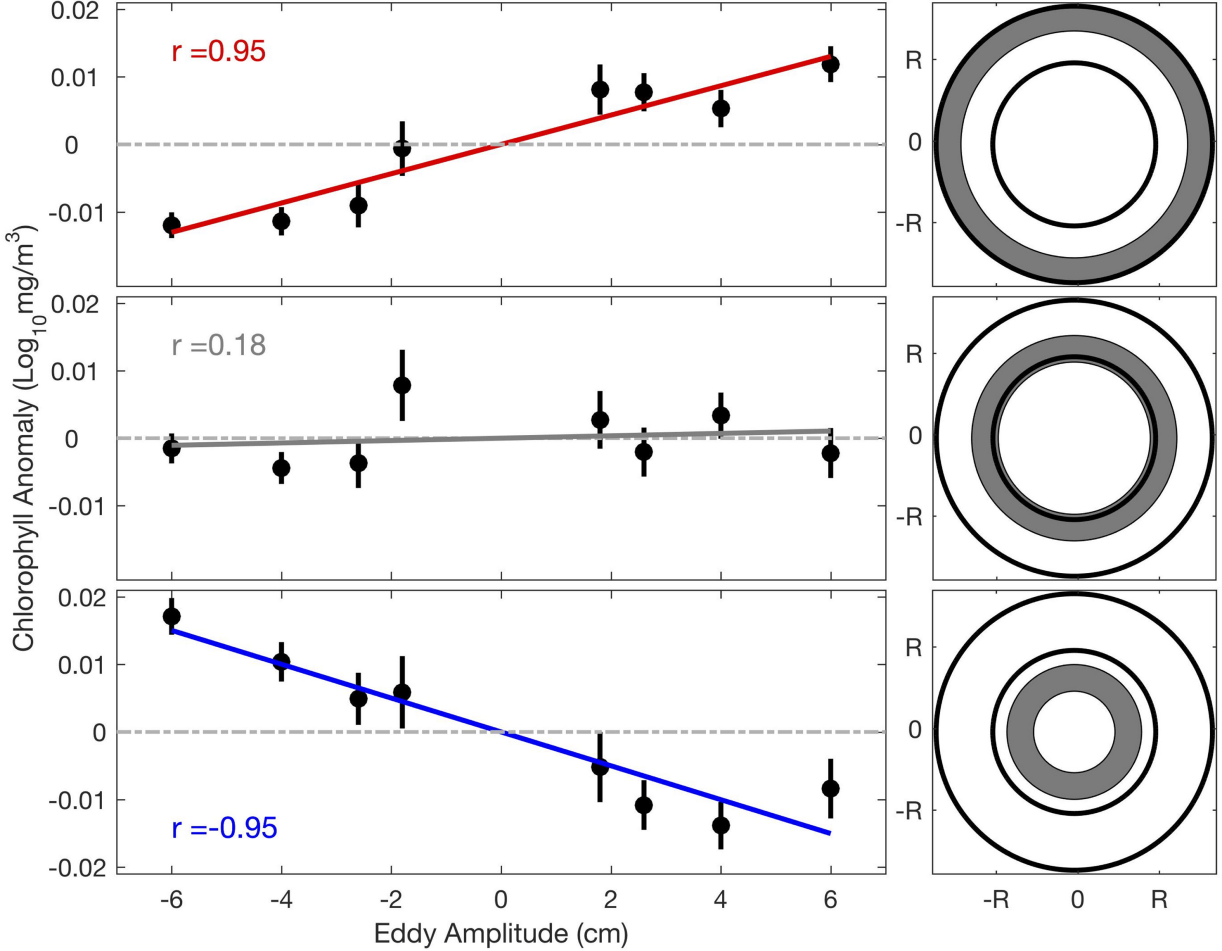
Linear regression for eddy amplitude and chlorophyll anomaly in different eddy area. Top: Linear regression within shaded area (the concentric ring with RDR = 1.55). Middle and bottom: Same as the top panels, except for the areas with RDR = 1.1 and RDR = 0.65, respectively. Only the eddies whose centroids are located within the area depicted in Fig 1A are used here. The negative (positive) amplitude represents cyclonic (anticyclonic) eddies. Short vertical lines show standard errors of chlorophyll anomaly for each bin. The correlation coefficient (r) of linear regression is shown in each left panel.

A case study to investigate the influence of eddies on chlorophyll distribution at different relative distance from the eddy core was further conducted in an area with 100-km radius and 800 km off Coquimbo (see Fig 1A for the location). The binned average of chlorophyll anomaly versus that of eddy amplitude is plotted in Fig 3 for different rings that were outside, along and inside the periphery, respectively. A linear fit was applied to quantify the impact of eddies on chlorophyll at different RDRs, and distinct features were found accordingly. For the region outside the eddy periphery (Fig 3, top panels), higher (lower) mean chlorophyll anomaly was generally found associated with anticyclonic (cyclonic) eddies. The eddies with larger amplitude were characterized by larger anomalous chlorophyll. The correlation coefficient, calculated by the Person method was applied to calculate, between amplitude anomaly and chlorophyll anomaly was 0.95, and the regression was significant at the 95% confidence level. Along the periphery (Fig 3, middle panels), the chlorophyll anomaly was not dependent on eddy amplitude, and the regression was not significant. For the region within the periphery of the eddies (Fig 3, bottom panels), the linear regression was significant with correlation coefficient of -0.95. Thus, in the interior and along the periphery of the eddies, higher (lower) mean chlorophyll anomaly was generally found to be associated with cyclonic (anticyclonic) eddies. Lags between eddy and chlorophyll were also tested, and the highest correlation was obtained without any lag.

**Fig 4:**
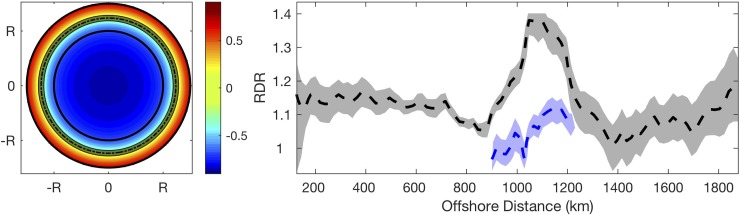
Schematic map for correlation coefficients and switching point off chile. Left: Schematic map describing the correlation coefficients of linear regression for concentric ring centered at different RDRs as illustrated in Fig 3. The dashed line represents the overall mean RDR where the correlation coefficient switches its sign with shading represents the linear regression not significant at the 95% confidence level. The black contours describe RDR = 1 and RDR = 1.55. The linear regression is significantly negative (positive) inside (outside) the shading region. Right: Dashed black curve depicts the switching point of correlation coefficients for eddies with different offshore distances. The grey shaded region represents the linear regression not significant. The blue curve and shading are calculated using the eddies with offshore distance between 900 and 1200km and between 30°S and 32°S. The linear regression is significantly negative (positive) below (above) the shading region.

Responses of chlorophyll anomaly to eddy amplitude varied inside and outside eddy periphery; and this feature was persistent off Chile. The impact of eddies on chlorophyll was further investigated for the different distances with respect to eddy center using all eddy snapshots to identify the transition zone where correlation coefficient switched sign from negative (inside) to positive (outside). For the concentric ring of RDR less than 1.2, higher (lower) chlorophyll anomaly was found to be associated with cyclonic (anticyclonic) eddies. On the contrary, the concentric ring of RDR larger than 1.2, lower (higher) chlorophyll anomaly was found to be associated with cyclonic (anticyclonic) eddies (Fig 4A). When the RDR of the concentric ring was close to 1.2, the correlation between chlorophyll anomaly and eddy amplitude was not significant and the corresponding RDR was identified as the transition zone.

The RDR for the transition zone varied with offshore distance of the eddy. To investigate the influence of offshore distance on location for the transition zone, correlation coefficient was calculated for eddies that were located at different offshore distance. To be specific, all snapshots falling into the 400-km wide meridional band were used to calculate the correlation coefficients to identify the transition zone. The center of the band was moving from the coast to offshore. The correlation coefficient generally changed its sign between RDR = 1.0 and RDR = 1.2, and the corresponding linear regression was not significant (Fig 4B). For the region between 1000 and 1200 km offshore, however, the reversal of linear regression did not happen until RDR was 1.3 or larger, indicating the chlorophyll anomaly inside and along the periphery of the cyclonic eddies was persistently larger than that inside and along the periphery of the anticyclonic eddies. To exclude the influence of islands and shallow topography (Fig 1A), the transition zone with offshore distance between 900 and 1200km was re-calculated using eddies between 30°S and 32°S where averaged bottom topography was deep with small variation (not shown). The RDR of the transition zone was around 1.1 (blue curve in Fig 4B), consistent with that in the other regions.

## 4. Discussion

The mesoscale eddies off Chile are investigated in this study. Like other Eastern Boundary Current Systems, the region is favorable for the formation of eddies [1]. The meander of the current and its interaction with topography can generate offshore jets and mesoscale eddies [5]. More eddies are generated at the leeward of major capes, e.g., Cape Coquimbo and Cape Concepcion. Also, the equatorward wind stress can drive offshore Ekman transport and upwelling near the coast, and the resultant instability can generate eddies [41]. The width of the shelf is so narrow along the Humboldt Current, where the shelf break is less than 200 km offshore [6], that most eddies propagate beyond the shelf without feeling the impact of the bottom. It is important to point out that these eddies may form even closer to the coast, but they can only be identified from satellite observations when they are more than 50 km off the coast. With improved observational systems, such as the upcoming sea water and ocean topography (SWOT) satellite, the altimetry observation near the coast will be greatly improved [42, 43].

Eddies play an important role in influencing the distribution of chlorophyll. Negative correlation between eddy amplitude and chlorophyll anomaly agrees with eddy pumping and trapping processes inside the periphery, but indicates weak eddy-Ekman pumping, which leads to opposite vertical transports and positive correlation [14]. The chlorophyll trapped by eddies will die off or be consumed by zooplankton in a short period [44], and the corresponding contribution to chlorophyll anomaly is less pronounced [21]. The persistent low chlorophyll within anticyclonic eddies is mainly caused by convergence transporting low-nutrient water into the eddy interior, and by downwelling depressing the nutricline [20]. The depth difference between mixed layer and nutricline varies spatially and temporally; thus, the nutrient content of upwelling in cyclonic eddies can change significantly [35]. As a result, the variance of chlorophyll level within cyclonic eddies is larger than that within anticyclonic eddies, and the corresponding values are more scattered for cyclonic eddies (not shown). Beyond the periphery of a cyclonic (anticyclonic) eddy, the sea level is higher (lower) compared to that of the eddy center, and the corresponding chlorophyll anomaly is negative (positive), being consistent with the negative cross correlation between sea level and chlorophyll anomaly off Chile [21].

The boundary of the eddy is highly dynamic where chlorophyll distribution can be impacted by the eddy. Chelton [3] mainly looked at regions where eddies propagated in the direction normal to chlorophyll gradient. They found a dipole feature was created around eddy periphery, and eddy stirring was stronger at the leading side than the trailing side. Off the coast of Chile, the zonal gradient of chlorophyll is much stronger than the meridional gradient (Fig 1A), and the eddies mostly propagate westward against the direction of chlorophyll gradient (Fig 1C). The flow on the southern (northern) side of the cyclonic (anticyclonic) eddy can advect higher chlorophyll offshore, and that on the northern (southern) side can advect lower chlorophyll onshore (Fig 2). The negative correlation between RDR = 1.0 and RDR = 1.2 (Fig 4A) indicates the integrated chlorophyll along the eddy periphery is larger for cyclonic eddies than for anticyclonic eddies. Therefore, the chlorophyll anomaly resulted from eddy pumping for anticyclonic eddies occupies a larger area with attenuated magnitude [35]. The horizontal advection superimposed on eddy pumping shifts the peak of negative chlorophyll anomaly closer to the periphery for the anticyclonic eddy (Fig 2).

The study mainly focuses on investigating the distances with respect to the eddy center, where the response of chlorophyll to anticyclonic eddies becomes larger than that to cyclonic eddies. The change of correlation coefficient from negative to positive happens at a narrow band, generally when near RDR = 1.15 (except between 1000 and 1200 km offshore, which will be discussed later). As RDR increases, the anomalous chlorophyll associated with anticyclonic eddies is higher than that associated with cyclonic eddies (Fig 4). The mixed-layer depth is elevated along and beyond the periphery of anticyclonic eddies compared to that at the center [28]. The nutrient-rich deep water is transported along the isopycnals to the periphery of anticyclonic eddies and stimulate the growth of phytoplankton near the surface [30]. The submesoscale processes and convergence (divergence) for cyclonic (anticyclonic) eddies outside the eddy periphery can drive opposite vertical transports compared with that inside the eddy [31]. Thus, the slope of linear regression turns positive beyond the eddy periphery.

For the region between 1000 and 1200 km offshore, the change of sign does not happen until RDR = 1.3 or so. This may be caused by the islands and shallow topography in the area. The land acts as a barrier to eddies; thus, the dynamic processes beyond the eddy periphery can hardly develop [33]. So, eddy pumping among other processes in the interior is predominate, and negative correlation between eddy amplitude and chlorophyll anomaly extends for larger RDR. Also, the shallow topography around islands results in elevated isopycnals that upwelling inside cyclonic eddies can transport more nutrients to the surface compared with that in the open ocean [35]. It is confirmed by calculating the transition zone using eddies that were not travelling near the islands (Fig 4B). The change of sign happens near RDR = 1.1, which is similar to other regions without influence of islands and shallow topography.

Eddies are predominately important for distribution of nutrients, and subsequent distributions of chlorophyll and biological production. But their impacts vary depending on background fields and dynamic processes. For example, the upwelling driven by eddy pumping inside cyclonic eddies brings nutrients to the surface and increases the new production [19]. On the contrary, the presence of eddies suppresses production in the Eastern Boundary Current Systems by transporting nutrients offshore [45]. The results shown in this study reveal the responses of chlorophyll outside and inside eddy periphery are quite different. Both cyclonic and anticyclonic eddies can enhance (depress) the level of chlorophyll at certain distance from the eddy center. This feature may lead to distinct impact on the marine ecosystem, which should be examined in future studies.

It is important to point out that the significant correlation between eddy amplitude and chlorophyll anomaly does not imply any direct causation. High correlation of linear regression does not necessarily indicate the relationship between eddy and chlorophyll being linear. Eddies with large amplitude are generally characterized by strong intensity [35]. However, eddies with small amplitude may have small sizes; thus, the intensity of eddy can also be strong. Concerning the process for eddy formation, the maximum amplitude is reached when the eddy is fully developed; however, the eddy already starts to decay at that point [21]. The amplitude of eddy may be impacted by the phase of eddy growth, topography, among others. As a result, amplitude is not the sole gauge for measuring the intensity of an eddy. Besides amplitude, other factors of eddies have also been checked for their influences on chlorophyll, such as polarity, radius and relative speed. All of them showed similar distance from eddy center where the responses of chlorophyll switch signs between cyclonic and anticyclonic eddies. Thus, itis a robust feature that the impact of mesoscale eddy on chlorophyll depends on the distances with respect to the eddy center.

## 5. Summary

In this study, we examined the influence of mesoscale eddies on the distribution of chlorophyll off the coast of Chile. By distinguishing the impact of mesoscale eddies on chlorophyll distribution at different distances with respect to the eddy center, we found cyclonic eddies induced more (less) chlorophyll in the center (surrounding area) than anticyclonic eddies. The higher responses of chlorophyll anomalies were generally associated with eddies that had larger amplitude. These results are important for improving our understanding of mesoscale dynamics. The distance with respect to the eddy center should be taken into consideration when investigating submesoscale/mesoscale transport and nutrient supply induced by eddies.

For the first time, the transition zone was defined and used to describe the location where the chlorophyll anomaly induced by anticyclonic eddies was larger than that by cyclonic eddies. The RDR for transition zone was generally around 1.15 for the eddies in our study area. For regions where eddies propagated near islands or over shallow topography, the RDR for the transition zone could increase to 1.3 or larger. Thus, impact of the mesoscale eddies is not localized or limited to the region within the eddies. This feature was robust off Chile regardless of filtering method or location. It would be of great interest to investigate the feature in other regions around the globe in future. An idealized model could help explain the submesoscale and mesoscale dynamics related with the processes.
